# Identification of Trail Following and Alarm Pheromones of *Lasius Flavus* Using Bioassay-Directed Fractionation

**DOI:** 10.1007/s10886-025-01651-w

**Published:** 2025-10-10

**Authors:** Thomas Butterfield, Jonathan Bacon, Elizabeth M. Hill

**Affiliations:** School of Life Sciences, University of Sussex, Falmer, Brighton, BN1 9QG England

**Keywords:** Mellein, Lasius flavus, 2,6-dimethyl-5-heptenol, Pheromone, Trail-following, Alarm

## Abstract

The identity of trail following pheromones secreted by the ant *Lasius flavus* were investigated using bioassay-directed fractionation techniques. Extracts of whole bodies were fractionated by high performance liquid chromatography and fractions tested for trail following activity in circular bioassays. Active fractions were analysed by gas chromatography- mass spectrometry (GC-MS) and structures of potential pheromones were confirmed by comparison with synthetic standards. The pheromones 2,6-dimethyl-5-heptenol (DMH) and mellein were identified in active fractions from extracts of whole body and headless ants respectively. Targeted analyses revealed that DMH was detected in the heads of worker ants at a concentration (mean ± SD) of 1.12 ± 0.58 ng per head. Mellein (stereoisomer not identified) was detected at 5.56 ± 1.4 pg per hindgut which was the lowest concentration of this compound detected in an ant species. In comparative bioassays of commercially available standards, (*R*)-mellein resulted in trail following activity at 0.01 pg per cm which is the lowest known detectable concentration of a pheromone by an ant, whereas a stereoisomeric mixture of DMH was 100-fold less active. These pheromones were tested for alarm activity by measurement of the mandible opening response in *L. flavus* worker ants. Standard DMH was found to cause a heightened level of aggression in at concentrations of ≥ 1 pg/l, indicating a likely function of an alarm pheromone. The response to (*R*)*-*mellein at the same concentrations was always lower, in keeping with its primary function of a trail following pheromone. This work reveals that bioassay directed fractionation can be a useful approach to identify the different behavioural pheromones in a social insect.

**Supplementary Information:**

The online version contains supplementary material available at 10.1007/s10886-025-01651-w.

##  Introduction

The social insects can encode complex information in multiple communication signals. Many ant species use mixtures of pheromones to communicate a complex variety of messages to their nestmates. Ants provide an ideal model for the study of pheromonal communication systems, as each species can produce a myriad of chemical signals, and their terrestrial lifestyle means their behaviours are easy to observe (Parry and Morgan 1979). A subset of the pheromones used by insects are known as recruitment pheromones and their purpose is to attract conspecifics to the source of the pheromone. There are two major uses of recruitment pheromones in the ants: to guide nestmates to a food source or nest site by laying trails of an attractive pheromone (a trail pheromone), and to attract nestmates to a threat (for example, a predatory intruder) by exuding an alarm pheromone into the air. This allows nestmates to assist the alarmed ant in dealing with the threat.

Previous work has identified the trail pheromones of over 60 species of ant, 16 of which are from the subfamily Formicinae (Morgan 2009). The trail pheromones of formicine ants have been particularly difficult to identify due to the relatively minute amounts of pheromone that they use; many myrmicine and ponerine ant glands contain 1-100ng of trail pheromone, whereas in the formicine ants this value tends to be less than 150pg (Morgan 2009). In all cases where a formicine species’ trail pheromone has been identified, it has been located in the hindgut; a small gland which forms the final part of the digestive tract and exits the gaster of the ant via the acidopore. All identified formicine trail pheromones are based on one of two potential chemical structures; either an isocoumarin structure or a δ-lactone structure (Morgan 2009). Of the 16 formicine ants with identified trail pheromones only two are in the genus Lasius, despite this genus being highly ecologically important within Europe (Czechowski et al. 2013; Vlasáková et al. 2009). In *Lasius niger* the trail pheromone was identified as 3,4-dihydro-8-hydroxy-3,5,7-trimethylisocoumarin (Bestmann et al. 1992) and in *Lasius fuliginosus* it was 3,4-dihydro-8-hydroxy-3-methylisocoumarin, known as mellein (Kern et al. 1997).

Both trail pheromones and alarm pheromones are known to cause attaction to worker ants. Alarm pheromones have been more widely studied than trail pheromones. This is primarily because they are present in more species (even non eusocial species can use alarm pheromones), but also because they are usually present in larger quantities than other pheromones (Blum 1969). Alarm pheromones in ants are often multicomponent and are produced and stored in a suite of glands. In formicine ants, alarm pheromones are generally stored and released from the mandibular gland, poison gland and Dufour gland. Alarm compounds in the mandibular gland most commonly have terpenoid structures such as citronellal; the poison gland often contains large amounts of formic acid; and the Dufour gland contains long chain hydrocarbons, most commonly undecane. Terpenoid compounds from mandibular glands often attract worker ants to the source of the pheromone (Wilson and Regnier 1971). In the genus Lasius, eight compounds, includng alkanes and monoterpenoids, have been described as alarm pheromones from six different species (Bergstrom and Lofqvist 1970; Regnier and Wilson 1968, 1969).

One member of the Lasius genus where the nature of recruitment pheromones has not been studied is the yellow meadow ant, *Lasius flavus*. This species of ant is ubiquitous throughout Northern Europe, and is the dominant ant species in meadow grassland (Czechowski et al. 2013). It is an ecosystem engineer which accelerates primary succession of grassland into forest (Vlasáková et al. 2009) changing the physiochemical properties of soils they inhabit (Boots and Clipson 2013; Wu et al. 2015). To date, trail pheromones in formicine ants have been present in the hindgut and preliminary work in our laboratory has confirmed that solvent extracts of hindguts of *L. flavus* do elicit a trail following response. However, the concentration of a chemical structure eliciting trail following activity is likely to be below 150pg in the hindgut of individual formicine ants, making detection of a relevant structure challenging (Morgan 2009). In this study, a novel bioassay- directed fractionation approach was used to purify and concentrate trace quantities of biologically active recruitment chemicals from large numbers of *L. flavus*. Extracts of ant bodies were fractionated by normal phase high performance liquid chromatography (HPLC). Fractions were tested for activity in a trail following bioassay, and chemical structures in active fractions investigated by gas chromatography-mass spectrometry GC-MS). Synthetic standards of putative pheromones were tested for both trail following activity and an alarm reponse in worker ants of *L. flavus.*

## Materials and Methods

### Chemicals

All solvents were HPLC grade and were purchased from Rathburn Chemicals Ltd, UK. *(R*) - mellein was purchased from Cayman Chemical and all other chemicals were purchased from Sigma Aldrich, UK.

### Collection and Storage of Ants

Eight colonies of *L flavus* were collected from the University of Sussex campus (Falmer, Brighton, UK) from undisturbed meadow grassland. Each colony and associated soil were housed in plastic tubs (30 cm x 45 cm x 25 cm) and kept in a natural day-night cycle at between 20 °C and 24 °C. When not being used for assays the colonies were fed twice weekly on mealworms (*Tenebrio molitor* larvae) and a 1:2 honey: water mixture was provided *ad libitum*.

### Extraction of Pheromones From Ant Workers

*L. flavus* workers were collected from a total of six laboratory colonies for pheromone extraction. In order to maximise the amount of trail pheromone collected, a foraging trail from each of the six colonies was set up to droplets of 1 M sucrose solution and ants were only collected from that trail. Ants were kept in a glass vial at −80 °C until extraction.

Either entire ant bodies (approx. 5300 ants) or headless ants (from approx. 4600 individuals) were ground for 3 min in a pestle and mortar in 40 ml diethyl ether. All the solid and liquid matter was sonicated with a probe (2 min) agitated with a vortex mixer (1 min) and centrifuged (3 min, 900 rcf). The supernatant was decanted to a glass vial and stored at −80 °C. The remaining bodies were extracted in the same way again and the supernatants combined, evaporated under oxygen-free nitrogen and redissolved in hexane (0.5 ml).

### Bioassay of Trail Following Activity

Ant extracts, HPLC fractions and test compounds were investigated for trail following activity using a modified circular bioassay (Evershed et al. 1982; Pasteels and Verhaeghe 1974). (Figure S1). The assay was printed onto standard printer paper and comprised 3 concentric circles divided into 10 arcs. Test extracts, dissolved in hexane (1 µl), were pipetted on the dashed circle between each arc (radii) which were 1 cm apart and laid in-between each of the ten radii marked around the circle, so the test trail was uniform and the total volume of test extract laid was 10 µl. An ant was placed in the centre of the bioassay in a small plastic enclosure. The hexane carrier solvent evaporated after approximately 15 s and the ant was released after 40 s. As the ant exits the inner circle it contacts the test trail and the number of radii crossed completely by the ant before it leaves either of the two bounding circles was counted. This was repeated on ten ants from a random selection of eight lab colonies. The median score of radii crossed was used to estimate of attractiveness of the test substance. Results for test extracts were tested against results for a hexane only control using Wilcoxon’s rank sum test.

### High Performance Liquid Chromatography Fractionation of Ant Extracts

Ant extracts were fractionated by normal phase HPLC using a Waters Spherisorb silica column (5 μm, 4.6 × 250 mm). Mobile phase A was 100% hexane and B 90% hexane/10% isopropanol with a 2 ml/min flow rate and a gradient of 100% A (5 min), to 10% B (35 min), 50% B (55 min) and 100% B (61–79 min). HPLC fractions were collected every minute and tested for trail following activity. Initially 10 µl aliquots from a group of every ten fractions (fractions 1–10, 11–20, 21–30 e.t.c) were combined and tested in the bioassay. When activity was detected in a group, 10 µl of each fraction comprising that group was diluted to 100 µl with hexane and then tested individually.

### Gas Chromatography-Mass Spectrometry Analyses of Fractions with Trail Following Activity

Fractions showing trail following activity plus their neighbouring fractions were analysed using a Thermo Trace GC Ultra gas chromatograph linked to a Thermo ITQ 1100 mass spectrometer. The carrier gas was helium at a flow rate of 1.3 ml/min and the GC column was an Agilent DB-5ms UI, 30 m x 0.25 mm x 0.25 μm film. Solvent in active fractions was evaporated to 3 µl under OFN and 2 µl was then injected into the GC-MS using splitless injection. The GC temperature programme was 60 °C for 4 min, to 300 °C at 10 °C/min, and held at 300 °C for 10 min. The mass spectrometer was used in EI mode (70 eV), with scanning range was between m/z 40 and 650. Chromatograms were analysed using Thermo Xcalibur software (Thermo Xcalibur 2.3 build 26) acquiring masses between 40 and 650 m/z. Compounds were tentatively identified by comparison with the NIST/Wiley database (NIST MS Search 2.0). Compounds were noted as potentially active if they were present in the fractions which produced a response in the circle bioassay and absent in neighbouring, non-active fractions. To confirm the identity of potentially active compounds, synthetic standards were analysed by GC-MS to confirm the retention time and mass spectra of the target compounds. If the identification was confirmed, the synthetic standards were then tested using the circle bioassay to ascertain if they were attractive to the ants.

### Preparation of 2,6-dimethyl-5-heptenol (DMH)

DMH was prepared by reduction of the corresponding aldehyde, 2,6-dimethyl-5-heptenal (97% purity, mixture of stereoisomers). The aldehyde was dissolved in 1 ml of a 1 M NaOH aqueous solution (1 mg/ml) to which sodium borohydride (NaBH_4_) (10 mg in 100 µl ethanol) was added dropwise. After 20 min at room temperature 10 drops of 2 M hydrochloric acid (HCl) were added to the reaction vial to decompose the NaBH_4_. DMH and its aldehyde was extracted by partition with hexane (1 ml). The yield was calculated by analyses of the concentration of aldehyde remaining after reduction using GC-MS analyses with citronellal (100 ng/µl) as an internal standard. Comparision with concentrations of the analyte with the internal standard revealed that 91.1% of the original amount of aldehyde was converted to the alcohol. The TIC signal for the alcohol in the post reaction solution was similar in intensity to 100 ng of the aldehyde standard confirming that most of the 2,6-dimethyl-5-heptenal was reduced to the corresponding alcohol.

### Quantification of Trail Following Pheromones in Ants

The concentration of identified pheromones were determined in ant heads and isolated hindguts of *L. flavus*. Three head extractions were made, each from a different colony and each containing the heads of five randomly selected foraging workers extracted in 10 µl of diethyl ether. Three hindgut extractions were also made from the same three colonies, each containing the hindguts of 20 randomly selected foraging workers extracted in 10 µl of diethyl ether. Internal standards (phenyl acetate and benzophenone, 0.5 ng/µl) were added to the extracts. The heads or hindguts were crushed in ether for 1 min, sonicated for 5 min and centrifuged. 2 µl of the supernatant from each extract was analysed by GC-MS using the same operating conditions as described above. Mass spectrometry data were collected using selected ion monitoring (SIM) or MS/MS modes (see Table S1 supplementary information). DMH was quantified with a calibration curve ranging between 0.5 and 6 ng on column using 0.5 ng phenyl acetate as an internal standard. Mellein was quantified using a calibration curve of 10–150 pg mellein and 0.5 ng benzophenone internal standard on column. Limits of detection were 10 pg (mellein SIM) and 500pg (DMH MS/MS).

### Behavioural Characterisation of Pheromones – Trail Function

Standard pheromones were assayed for trail following behaviour in the circle assay. The compounds were tested at concentrations between 1 fg/µl and 100 ng/µl. Hexane was used as a negative control and a hindgut extraction containing 10 hindguts in 100 µl hexane was used as a positive control.

### Behavioural Characterisation of Pheromones – Alarm Function

Pheromone standards were tested for alarm function using the Mandible Opening Response (MOR) assay. This is an established assay used to test for an alarm response of ants to a test compound by detecting a mandible opening behaviour (Guerrieri and d’Ettorre 2008). 20 ants, randomly chosen from 6 lab colonies, were harnessed in plastic pipette tips using masking tape ensuring that only the head of the ant protruded from the tip of the pipette. The identity of each ant was recorded for use in statistical analysis. The ants were left for 2 h to habituate to the harnesses. 1 µl of a solution of each test compound in hexane was applied to a 2 mm x 2 mm square of filter paper and left for 25 s to allow the solvent to evaporate. The square was presented to a test ant at a distance of 1 mm from the antenna and care was taken not to touch the antenna of the test ant. The compounds were tested in the following concentrations: 1 pg/µl, 10 pg/µl, 100 pg/µl, 1 ng/µl, 10 ng/µl and 100 ng/µl. Hexane was used as a negative control and a whole body of a non-nestmate worker was held in forceps 1 mm away from the test ant antennae as a positive control. The latter was used to expose test ants to the natural mixture of alarm pheromones that they would encounter in the wild, as ants display full alarm behaviour when grasped by forceps. The controls and treatments were presented to the same set of 20 test ants in a randomised order.

### Statistical Analysis

All circle assay data was analysed used Wilcoxon rank sum tests as the data were not normally distributed. Ants were tested repeatedly with different treatments for the MOR assay, and the data analysed using generalized linear mixed models (GLMM). The model included individual ant worker nested within colony as a random factor to account for individual variation of ant response in the analysis. The maximal model contained test compound concentration, compound tested and test order as fixed factors. As the MOR assay produces a binary response variable, treatments were compared using a binomial distribution and a logit-link function (the canonical link function for binomial response models). All factors were used in the final model, as there were no non-significant variables to remove. All statistical analysis was performed in R v3.1.3 (R Core Team (2015).

## Results

### Trail Following Activity of Extracts of L. Flavus

**Fig. 1:**
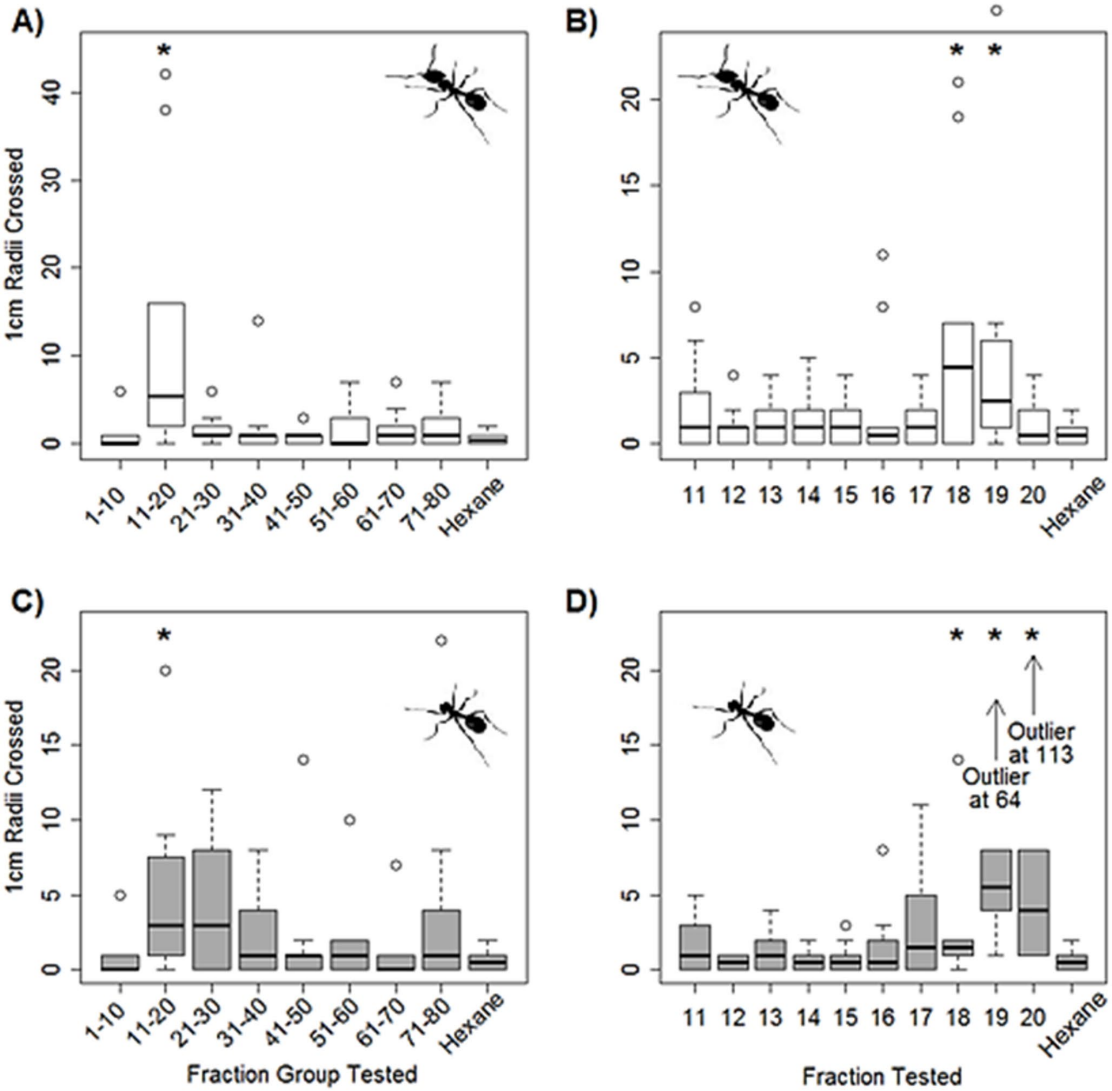
Activity of HPLC fractions in the trail following assay. **A** Groups of fractions from whole ant extracts, **B** Individual HPLC fractions from whole ant extracts, **C** Groups of fractions from extracts of headless ants, **D** Individual fractions from extracts of headless ants with outliers at 64 and 113 radii crossed when fractions 19 and 20 were tested respectively. Bars show median values, boxes show interquartile ranges, whiskers show extreme ranges and outliers are displayed as open circles. The * symbols show test extracts that elicited a response significantly higher than the hexane control (*p* < 0.05)

Whole body extracts from ants were separated by HPLC and groups of fractions collected and tested for activity in the circular trail following assay. The only group of fractions which produced a trail following response significantly higher than the hexane carrier control was the group eluting between 11 and 20 min (Fig. 1A) (W = 84, *p* < 0.01). When each of these fractions in this group were tested individually, fractions 18 and 19 produced a trail following response that was significantly higher than the hexane control (Fig. 1B) (fraction 18: W = 23, *p* < 0.05, fraction 19: W = 14.5, *p* < 0.01). The median number of radii crossed by test ants for fractions 18 and 19 was 4.5 and 2.5 respectively.

The experiment was repeated with extracts of headless ants which were fractionated by HPLC. Again, the group containing fractions 11–20 was the only group to elicit a trail following response significantly greater than the hexane control (Fig. 1C) (W = 90.5, *p* = 0.01). When the 11–20 group of fractions was reanalysed without the outlier, the trail following response was still significantly higher than the control (W = 63.5, *p* = 0.03). When tested individually, fractions 18, 19 and 20 all produced a significantly greater following response than the hexane control (fraction 18: W = 21.5, *p* < 0.05; fraction 19: W = 3, *p* < 0.001; fraction 20: W = 9.5, *p* < 0.01) (Fig. 1D). The median radii crossed by ants when fractions 18, 19 and 20 were tested was 1.5, 5.5 (excluding an outlier at 64 radii crossed) and 4 (excluding an outlier at 113 radii crossed) respectively. The median response to a hexane control was never above 1 crossed radius.

External standards were run on the HPLC prior to fractionation of the samples to monitor any shifts in retention time of the fractions. The activity profiles for Fig. 1B and D (the individual fractions for whole body and headless extractions respectively) are directly comparable, as the external standards run on the HPLC instrument prior to separation of the extractions had the exact same retention times. The most potent behavioural response to the whole body fractions was in fraction 18 (4.5 median radii crossed), whereas the most potent from the headless extraction separation was fraction 19 (5.5 median radii crossed). The median response to fraction 18 in the headless extraction was just 1.5 radii crossed. This indicated that an attractive compound in the head eluted from the HPLC at 18 min which was absent from the headless extraction.

### Identification of an Active Compound From Whole Body Extraction of Ants

**Fig. 2:**
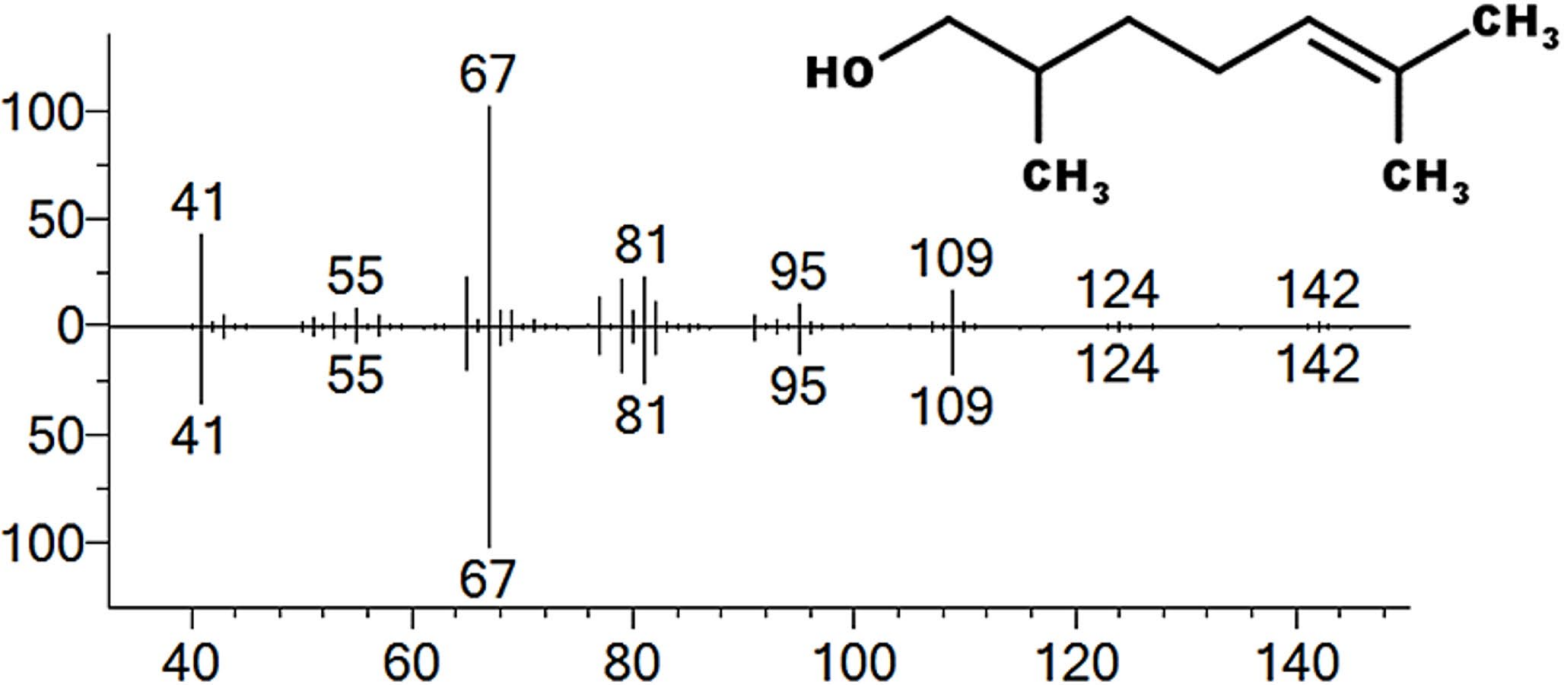
Electron ionisation mass spectrum of the compound of interest present in fractions 18 and 19 from whole body extracts of ants. Plot of percent ion abundance against m/z. Top plot: the mass spectrum for the peak at GC retention time 9.93 min which was identified as 2,6-dimethyl-5-heptenol (DMH). Bottom plot; the mass spectrum of a synthetic standard of DMH

HPLC fractions containing trail following activity as well as neighbouring in active fractions were analysed by GC-MS. Examination of the total ion chromatograms revealed one peak eluting at a retention time (RT) of 9.93 min which was present in active fractions 18 and 19 but absent in inactive fractions 17 and 20 (Figure S2 supplementary information). Analysis of the mass spectrum revealed ions at m/z 109, 95, 67, 55 and 41 corresponding to loss of H_2_0 and alkyl groups from a molecular ion of m/z 142 (Fig. 2). This spectrum was found to match that of 2,6-dimethyl-5-hepten-1-ol (DMH) (Law et al. 1965). GC-MS analysis of a synthetic standard of DMH confirmed an exact match of the retention time and mass spectrum for this compound.

**Fig. 3:**
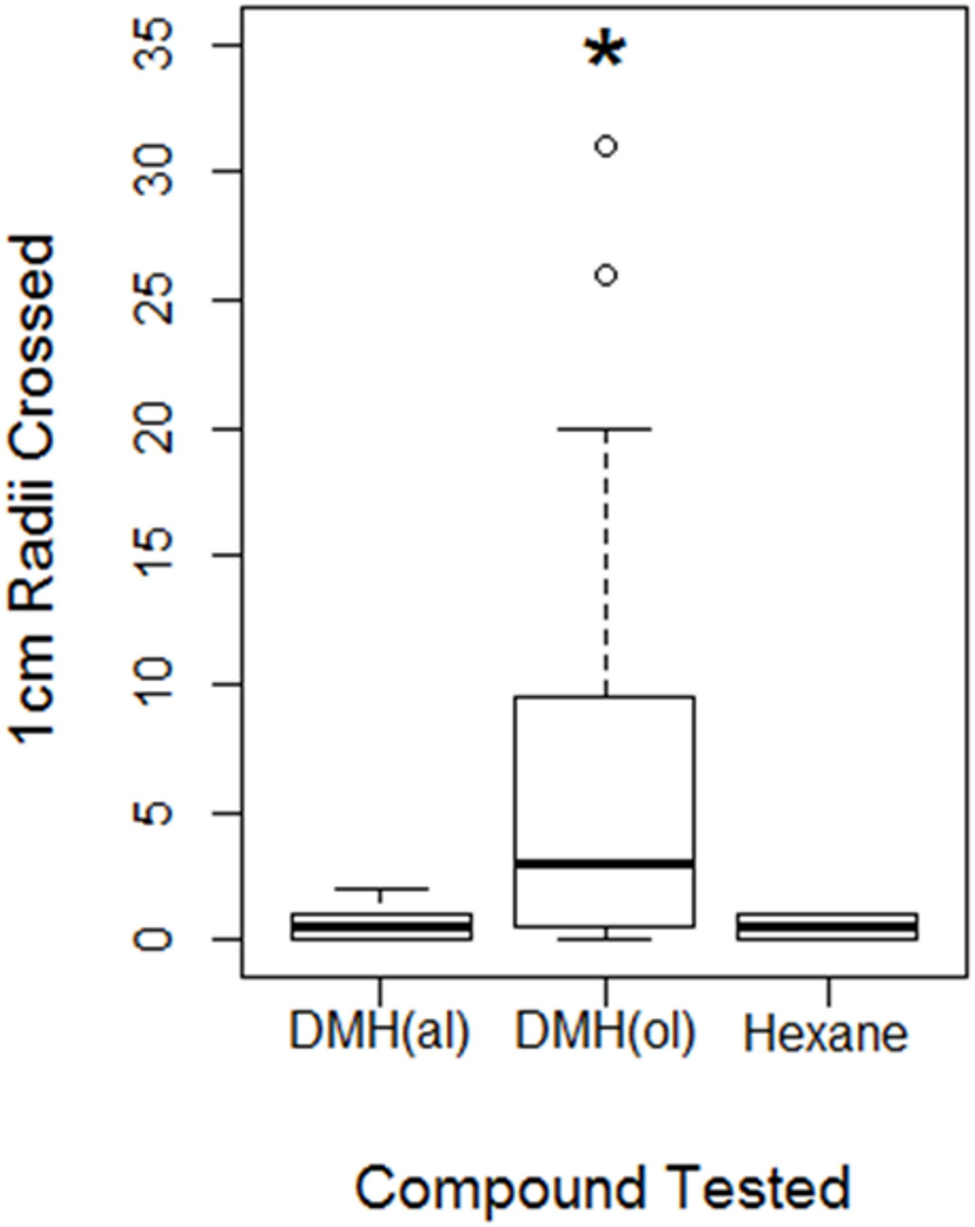
Trail following assay of DMH

Standard DMH and the corresponding aldehyde (both were mixtures of stereoisomers) were tested in the trail following assay at a concentration of 1 ng/l. DMH elicited a significantly greater trail following response compared with a hexane control (W = 45, *p* = 0.014) with 3.5 median radii crossed compared with 0.5 radii for the hexane control. The equivalent aldehyde, 2,6-dimethyl-5-hepten-1-al, did not produce a trail following response (0.5 median radii crossed) revealing the importance of the alcohol functional group for trail following activity (Fig. 3).

The median number of circle assay radii crossed by test ants when DMH as the alcohol (DMH(ol)) and the equivalent aldehyde, (DMH(al)) were compared with a hexane control. Compounds were tested at a concentration of 1ng/µl. The bars show median values, boxes the interquartile range, whiskers the extreme range and outliers are displayed as open circles. The star designates that the response to DMH(ol) was significantly greater than to a hexane control (*p* = 0.014).

### Identification of an Active Compound From Headless Extractions of Ants

Recruitment pheromones can be present in both the head and abdomen of ants and DMH has previously been identified as a pheromone present in mouthparts of *L. claviger* (Regnier and Wilson 1968). To determine which compounds with recruitment activity were present in the fractionated extracts of headless ants, active fractions were investigated by GC-MS. The fractions which produced the highest trail following activity in the circle assay were fractions 19 and 20 (Fig. 1D), and these plus neighbouring fractions 18 and 21 were analysed using GC-MS. Examination of the total ion chromatograms did not reveal obvious peaks peculiar to the active fractions alone. However, several ions were detected in fraction 19 including m/z 178. The ion chromatogram of m/z 178 in fraction 19 (the most active) revealed a compound eluting at 15.92 min which was absent in other fractions (Figure S3). No other peaks were identified as potential recruitment pheromones, as they were all either present in the non-active or weakly active fractions, or absent in fraction 19 (the most active).

**Fig. 4:**
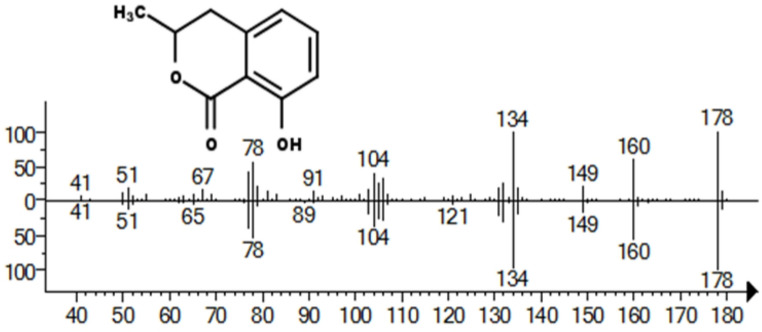
Electron ionisation mass spectrum of the compound of interest present in fraction 19 from extracts of headless ants. Plot of percent ion abundance against m/z. Top plot: the mass spectrum for the peak at GC retention time 15.92 min which was identified as the dihydroisocoumarin mellein. Bottom plot; the mass spectrum of a synthetic standard of mellein

GC-MS analysis of the peak at 15.92 min revealed ions at m/z 178 (M^+^), m/z 160 (loss of H_2_O from M^+^) and m/z 149 and 135 (arising from cleavage of the lactone ring) (Fig. 4). The compound was identified as the dihydroisocoumarin, mellein, and confirmed by analysing a synthetic standard of mellein which revealed an identical retention time and mass spectrum.

**Fig. 5:**
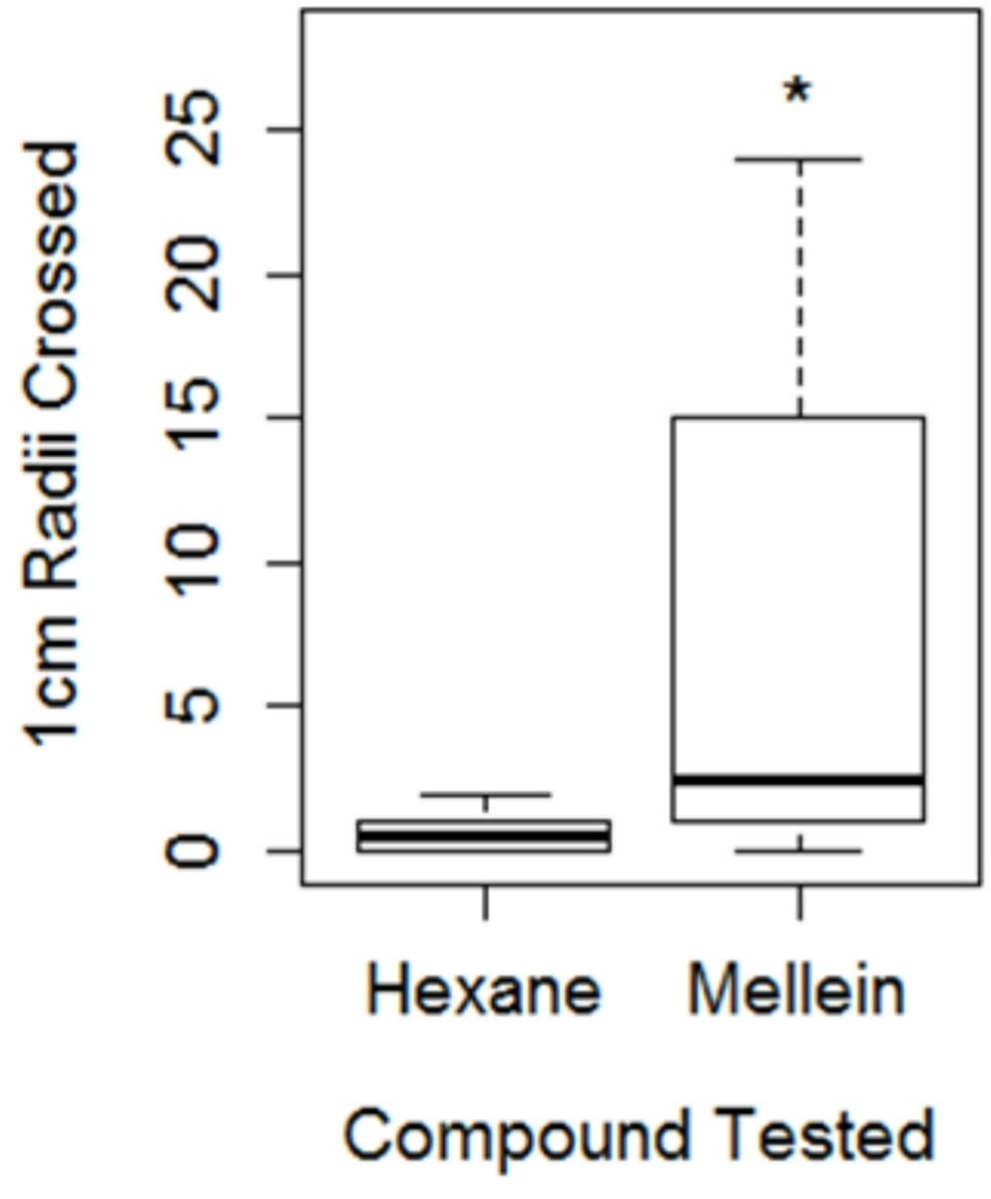
Trail following activity assay of mellein (*R* form)

In our study, the stereochemistry of mellein extracted from the ants was not determined, and the enantiomers would not have been separated on the GC column. We used a commercially available standard of mellein (*R* form) to test for trail following activity. When tested in the circle assay, ants followed (*R*)-mellein (1 ng/µl) significantly further than they did a hexane control (W = 76, *p* = 0.044) (Fig. 5). The median response of ants to hexane was 0.5 radii crossed, whereas the median response to mellein was 2.5 radii crossed. The trail following activity of the *S* enantiomer was not determined.

Bars show the median response, boxes the interquartile range and whiskers the extreme range. The star indicates the response to mellein was significantly greater than the response to a hexane control (*p* = 0.044).

### Quantification of Pheromones in Extracts of Ant Heads and Hindguts

To identify the source of the pheromone compounds, three repeat extractions were made of the. ant heads and hindguts which were the location of trail pheromones in other formicine ants (Morgan 2009). To increase sensitivity, analytes were detected by SIM (internal standards and DMH) or MS-MS (mellein). Pheromones were quantified using calibration curves (R^2^ > 0.998). Mellein was found only in hindgut extractions at a mean ± SD concentration of 5.56pg ± 1.4pg per hindgut and DMH was found only in head extractions at an average concentration of 1.12ng ± 0.58ng per head.

### Comparison of Trail Following Response to Pure Standards of Identified Pheromones

**Fig. 6:**
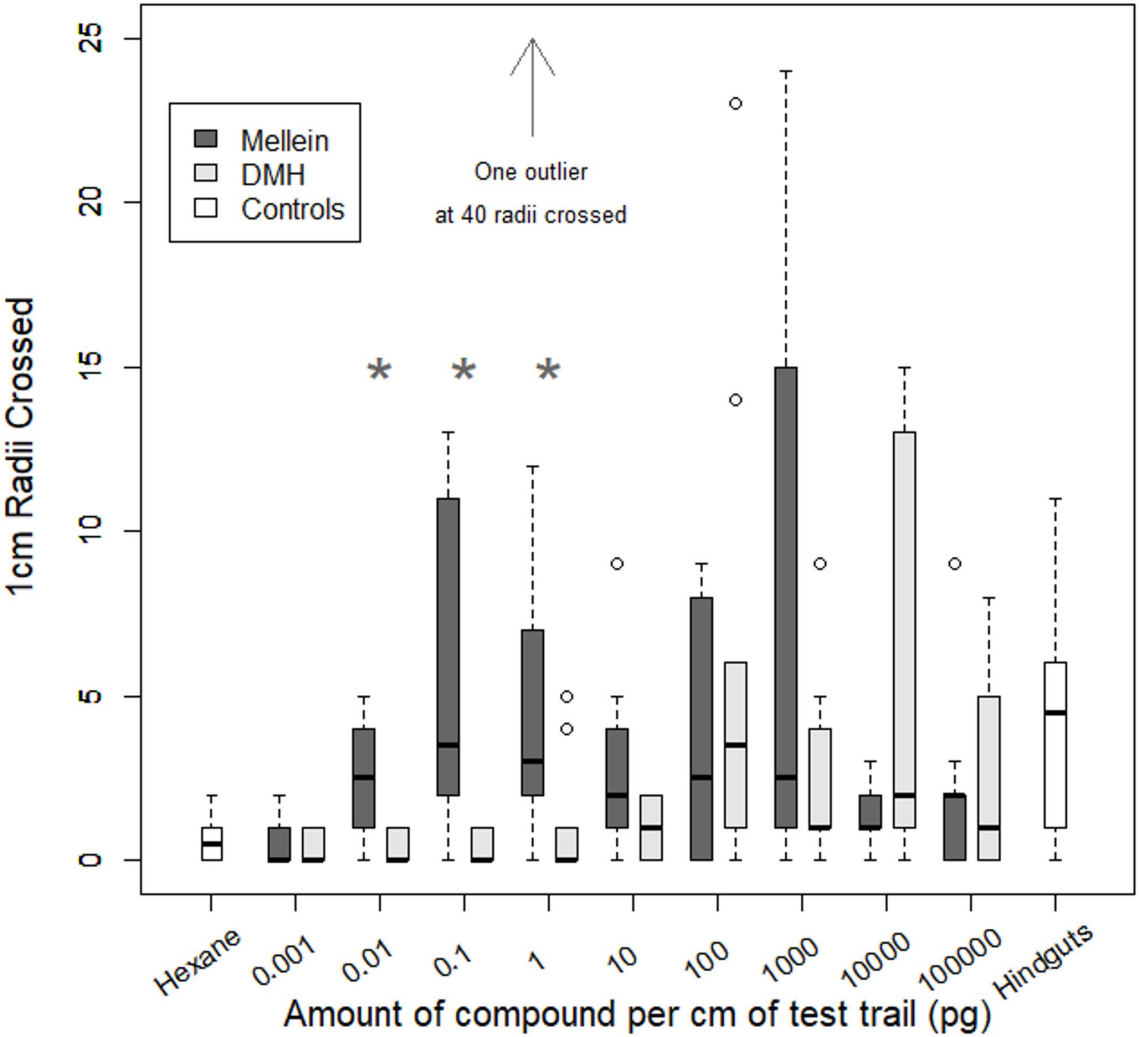
The trail following response to pheromone standards. The data show medians, interquartile ranges and extreme ranges with outliers displayed as circles. Ants followed mellein further than they did DMH and behaviour was elicited at far lower concentrations of mellein. The stars indicate that the response to mellein was significantly higher than the response to DMH (*p* < 0.05)

The circle assay was used to test the potency of synthetic standards of the two trail following pheromones. DMH and (*R*)- mellein were tested at a range of concentrations alongside a negative control of pure hexane and a positive control of a hindgut extraction. All test solutions were applied at a rate of 1 µl per 1 cm arc of the assay circle. The lowest concentration of mellein for which test ants showed a significant trail following response was 0.01 pg/µl (*p* < 0.03) (Fig. 6). Mellein concentrations up to 1000 pg/l revealed a significant trail following response, however these responses did not show a clear dose-response relationship as the median arcs crossed stayed approximately constant with increasing concentration. No significant trail following activity was observed at the highest test concentrations of 10 and 100 ng/µl mellein (*p* > 0.05).

The response profile of DMH was very different to that of mellein. Significant trail following activity was only detected at concentrations of 100 pg/µl and 10 ng/l (*p* < 0.01). Again, there was no clear dose-response relationship present. At the same concentrations, mellein showed a longer trail following response than DMH at levels lower than 100 pg/µl This difference between the test compounds was significant when the concentrations were 0.01 pg/µl (*p* < 0.01), 0.1 pg/µl (*p* < 0.005) and 1pg/µl (*p* < 0.05). There were no significant differences between the responses to the two test compounds at any other concentration.

The hindgut extraction used as a positive control in the circle assay comprised 10 hindguts in 100 µl hexane and showed significant trail following activity (W 18, *p* < 0.01). Previous quantification indicated that the concentration of mellein in this hindgut extraction would be 0.56 pg/µl (5.56 pg/hindgut). The median response by test ants to the hindgut extraction was 4.5 radii crossed (Fig. 6). The approximately equivalent amount of mellein tested (1 pg/µl) resulted in 3.5 median radii being crossed. Comparison of data from the circle assay revealed that the responses to hindgut extraction and to 1 pg/µl mellein were not significantly different (W = 50.5, *p* > 0.05,) indicating that mellein may account for all the trail following behaviour present in the hindgut extraction.

### Alarm Activity of Standards of Identified Pheromones in the Mandible Opening Response (MOR) Assay

The mandible opening response assay was used to test whether either of the identified pheromones were likely to be an alarm pheromone based on the observations that many species of ants open their mandibles when alarmed (Blum 1969). DMH and (*R*)-mellein were tested at concentrations between 1 pg and 100 ng per 1 µl of test solution applied to the test filter paper. A negative control of pure hexane and a positive control of a live and alarmed ant were also tested. The response rate is expressed as a percentage of 20 test ants which showed a MOR.

**Fig. 7:**
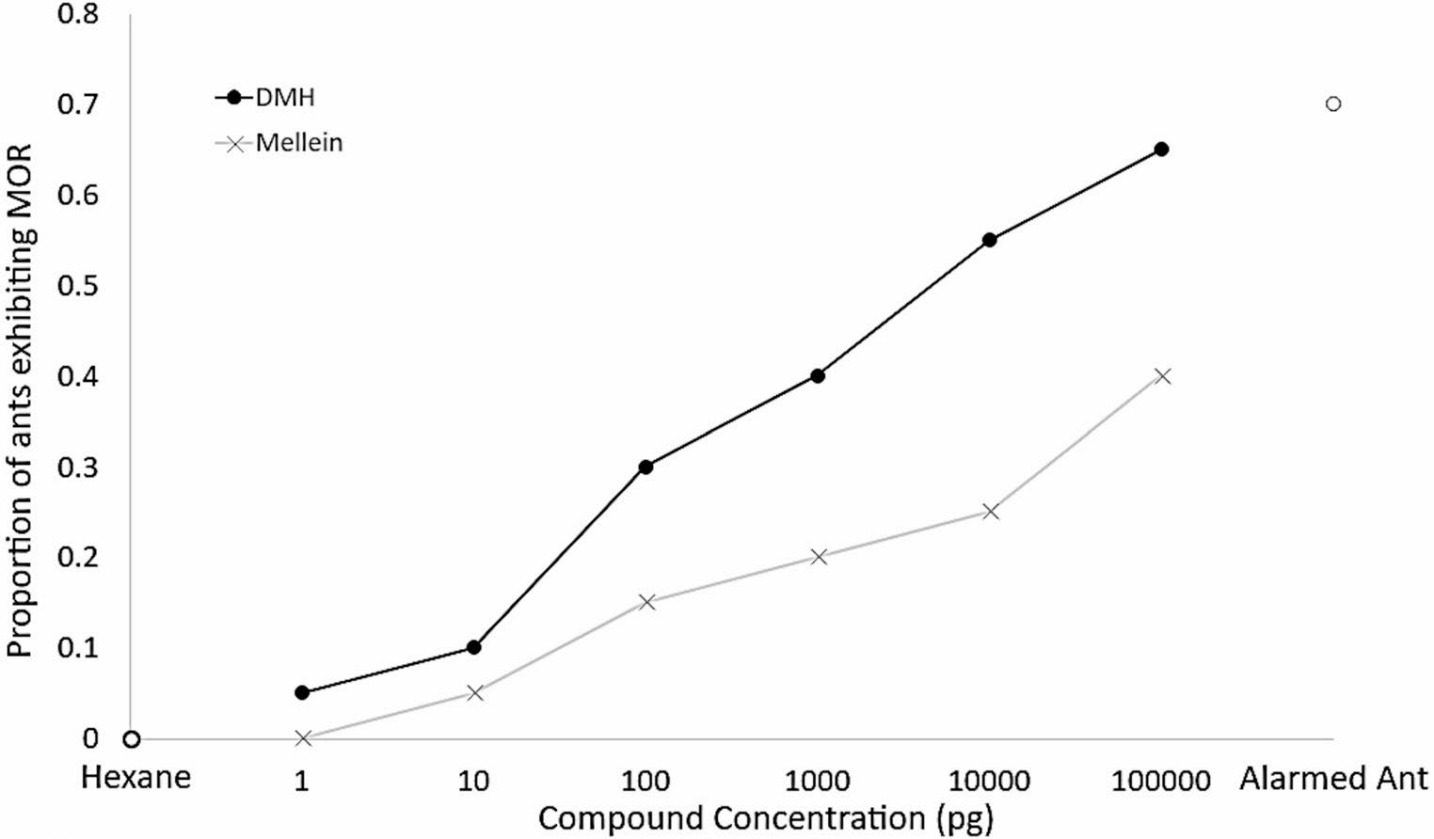
The activity of standard recruitment pheromones assayed in the mandible opening response. 20 worker ants were tested under exposure to either DMH, (*R*)-mellein, hexane or a non- nestmate alarmed ant (o). DMH and mellein were tested at a range of concentrations. A higher proportion of ants exhibited the mandible opening response when exposed to DMH than they did to mellein

When mellein was used as the test substance, a mandible opening response was first detected at 10 pg/µl and the response rate was 5% of test ants exhibiting a MOR (Fig. 7). With increasing concentrations there was a clear dose response relationship, with the response rate gradually increasing with increasing concentration of mellein. The maximum response to mellein in the assay reached was at the highest test concentration of 100 ng/µl with 40% of test ants displaying a response. When DMH was tested in the MOR assay, a response was first detected when the test concentration was lowest at 1 pg/µl, and the response rate was 5%. Again, a dose response relationship was present, and the response rate increased to 65% at the highest test concentration of 100 ng/µl. Analysis of the data in the GLMM model revealed that the proportion of ants which exhibited the MOR was always significantly higher when presented with DMH than with mellein, no matter the test concentration (χ2 = 6.285, *p* = 0.012). The highest overall response rate occurred when a live, alarmed ant was presented to test ants, where 70% of ants displayed the MOR. Hexane produced a 0% response rate.

Given the quantitation of DMH concentrations in extracts of ant heads, the maximum amount of DMH emitted by an alarmed ant used as a positive control in Fig. 7 cannot have been more than 1.12ng. The rate of MOR responses for non-nestmate ants was 70%, and when compared to the approximate equivalent amount of pure DMH (1 ng) the response rate was 40%. Although a two-proportion z test did not find a significant difference between these two values (*p* > 0.05, χ^2^ = 2.53), it was possible that DMH may not be the sole component of the *L. flavus* alarm pheromone as the response rate to pure DMH did not reach the same level as when a live, alarmed ant was used as the stimulus.

## Discussion

Using a novel combination of bioassay-directed HPLC fractionation of ant extracts followed by GC-MS analyses of fractions containing trail following activity, two recruitment pheromones of the yellow meadow ant, *L. flavus*, were identified. This approach allowed the identification of trace quantities of the pheromone, mellein, despite it being present at minute amounts of 5 pg per hindgut. Their behavioural functions have been compared and their glandular sources identified.

The pheromone identified compound in the ant heads, DMH, is a terpenoid compound with a terminal hydroxyl group. It has previously been identified in the heads of *L. claviger* (previously *Acanthomyops claviger*) males (Law et al. 1965) who hypothesized that it may be a sex pheromone discharged during the mating flight to attract the queen. However, DMH has also been detected in sterile workers of *L. claviger* which showed a strong alarm response to the pure standard (Regnier and Wilson 1968). In our study, DMH was detected in heads of *L. flavus* workers and is likely to be stored in the mandibular gland which is the major gland present in the heads of formicine ants. In the MOR assay, exposure to a pure standard of DMH at concentrations between 1 pg and 10 ng resulted in a dose dependent aggression response. These findings suggest that DMH is an alarm pheromone of *L. flavus*. However, the concentration of DMH in *L. flavus* workers was 1.12 ng per head and this concentration elicited a MOR response of 40% which was lower than the 70% response when an alarmed non-nestmate worker was used as the stimulus, although this difference was not statistically significant. This finding may indicate that DMH is likely to be a component of a multicomponent alarm pheromone, as is the case for many other ant species (Regnier and Wilson 1968). Alternatively, the effect of other stimuli contributing to the alarm response such as the visual stimuli of alarmed ants, or the response from pheromone being sprayed from the mandibles rather than volatilising from filter paper, cannot be ruled out.

Some pheromones have been shown to exhibit both alarm function as well as recruitment activity (Hölldobler et al. 2013). This dual function serves to attract nestmates to a threat, for example a predatory intruder, by exuding an alarm pheromone with recruitment activity into the air. In our study, DMH was also active to *L. flavus* workers in the trail following circle assay although it was 1000 fold less potent compared to mellein. Mellein is a dihydroisocoumarin compound which has been previously identified as the trail pheromone in the congeneric ant species *L. fuliginosus* (Kern et al. 1997), and in another formicine species, *Formica rufa* (Morgan 2009). Mellein has also been found in the hindguts of three other formicine ants: *F. fusca*,* Camponotus silvicola and C. rufipes*, but did not elicit trail following behaviour in conspecific species (Morgan 2009). Likewise, our study detected mellein in the hindguts of worker ants, however it produced a very strong trail following response in the circle assay of conspecific ants even at low (sub-picogram) concentrations. This finding strongly suggested that mellein is a potent trail pheromone in *L. flavus*. In our study, a hindgut extract produced a response of 4.5 median radii crossed by test ants in the trail following assay and would have contained 0.56 pg/µl mellein. The closest test concentration of a standard of pure (*R*)-mellein (1 pg/µl), resulted in a median response was 3.5 radii crossed in the same assay. A Wilcoxon rank sum test found no significant difference between the two responses, and the interquartile ranges shown in Fig. 6 clearly overlap. This indicated that mellein may account for all the trail following activity which is elicited by response to a hindgut extraction and suggested that it is the primary trail pheromone of *L. flavus.* Furthermore, as mellein elicited trail following at a concentration of just 0.01 pg/µl, it appears to be present in the hindgut at a concentration 50 times higher than is necessary to produce trail following behaviour. However, it should be recognised that the stereochemistry of extracted mellein and the trail following activity of *S* form of mellein still needs to be determined.

Compared to the other two species in the *Lasius* genus where trail pheromones identified, the amount of mellein detected in *L. flavus* was significantly lower. *L. niger* hindguts contain 150–200 pg of mellein (unpubl data our lab) and *L. fuliginosus* hindguts contain 50-100pg (Kern et al. 1997). *L. flavus* hindguts only contained 5.56pg ± 1.4pg of mellein, which was tenfold less than the lowest estimate in *L. fuliginosus*. To our knowledge this is the lowest concentration of trail compound ever identified from an ant. *L. flavus* were also extremely sensitive to their trail pheromone and were able to detect it at a trail concentration of 0.01 pg/cm. The leaf cutting ants *Atta sexdens sexdens* could detect and follow their trail pheromone at a concentration of 1.5 pg/cm of trail, but this was still 150x less sensitive than *L. flavus* (Morgan et al. 2006). Very few studies have investigated how sensitive ants are to their trail pheromones, but *L. flavus* appears to be the most sensitive to date. In general, compounds are tested in nanogram quantities, which is biologically relevant if the species in question possesses similar quantities in their glands (Evershed et al. 1982; Jackson et al. 1990). Other *Lasius* species may also be highly sensitive to extremely low concentrations of their trail pheromone substances, but this remains to be investigated. The low abundance of trail pheromone combined with the high sensitivity of workers may be an adaptation to the subterranean lifestyle of *L. flavus*. An unusual life history trait of *L. flavus* is that the workers forage almost entirely underground and not on the surface like other congeneric species such as *L. niger.* This subterranean lifestyle means that foraging workers are exposed to a much more restricted air flow than surface foraging species. Along with the generally cooler temperatures found underground, this may mean that any chemicals deposited by the ants are likely to evaporate much more slowly from the environment. As a result, it is possible that pheromones produced by this species are present in much lower quantities compared to species that forage primarily above ground. This suggests that *L. flavus* can reduce the amount of energy they expend on trail pheromone production by only laying very small amounts in trails.

Mellein was identified in fraction 19 from HPLC fractionation of extracts of headless ants. The compounds responsible for the trail following activity detected in other neighbouring HPLC fractions (fractions 18 and 20 in Fig. 1D) have not been identified. However, if mellein was present in these fractions, it could cause trail following activity at sub-picogram levels which would not be detectable by GC-MS analyses. Although the stereochemical structure of mellein detected in *L. flavus* has yet to be determined, the (*R*)-mellein enantiomer was clearly active as a trail following pheromone to workers of *L flavus*. In a previous study it was reported that the trail-following activity *in L. fuliginosus* by (*R*)-mellein was significantly higher than that elicited by its (*S*)-enantiomer, or the racemic mixture (Kern et al. 1997).

An important consideration of the findings presented in our study is the similarity of behavioural responses when presented with an alarm and a trail pheromone. Both types of pheromone elicited some level of trail following and alarm activity. It was only by comparing the behavioural responses to both pheromones in each assay that their functions were elucidated. This process has not been routinely performed in previous published studies. Had either of the pheromones identified in this work been tested using either assay in isolation, misidentification of their primary function could easily have occurred. For this reason, the comparative examination of responses to putative pheromones in a range of concentrations in different bioassays should always be considered.

The methodology used in this study should allow for further advancement in the field of recruitment pheromone identification. The detection of trace amounts of biologically active molecules in extracts of ants is a challenge. The use of a bioassay-directed fractionation approach allows purification and concentration of pheromones in samples which facilitates their identification by mass spectrometry. Furthermore, this experimental method is not limited only to ants, but any other organism where sufficient quantities of material are available, and which can be tested in relevant bioassays.

## Supplementary Information

Below is the link to the electronic supplementary material.

ESM 1 (DOCX 252 KB)

## Data Availability

Data is provided within the manuscript or supplementary information.
