# Supplementary material for: Identification of Trail Following and Alarm Pheromones of Lasius Flavus Using Bioassay-Directed Fractionation
Source: J Chem Ecol. 2025 Oct 10;51(5):99. doi: 10.1007/s10886-025-01651-w (PMC12513911; doi:10.1007/s10886-025-01651-w)
Supplement: Supplementary file 1 — (DOCX 252 KB) [file 10886_2025_1651_MOESM1_ESM.docx]

**Supplementary information.**

| **Target Compound** | **Time (mins)** | **Method** | **Masses Monitored** |
| --- | --- | --- | --- |
| Phenyl acetate (internal standard) | 8 - 9 | SIM | 51, 66, 94 |
| 2,6-dimethyl-5-heptenol (DMH) | 9.6 - 11 | SIM | 67, 81, 109, 142 |
| Mellein | 14 - 16.5 | MS/MS | 178 (parent): 160, 134 (daughters) |
| Benzophenone (internal standard) | 16.51 - 17 | SIM | 77, 105, 182 |

**Table S1. Selected ion monitoring (SIM) and MS/MS conditions for the quantitation of identified pheromones and internal standards.**

**
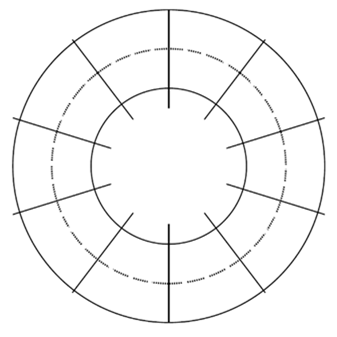
**

**Figure S1**. **The trail following bioassay.** Three concentric circles divided into 10 arcs were printed onto standard paper. Test extracts (1µl) were placed on the dashed circle between each arc which were 1 cm apart.

**
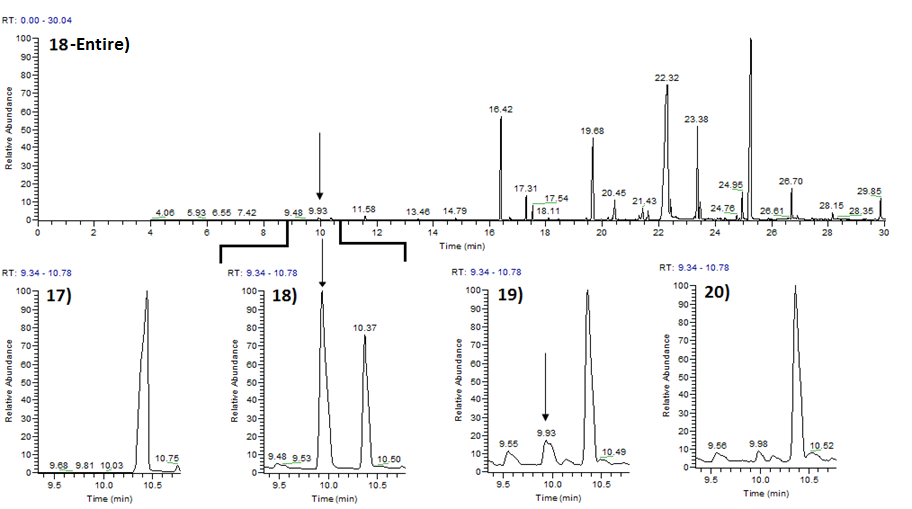
**

**Figure S2.** **GC-MS analyses of HPLC fractions from whole body extracts of ants.**

Total Ion Chromatograms of HPLC fractions numbered 17-20. GC-MS analyses revealed a compound of interest with a retention time of 9.93 mins which is marked by the arrow. This compound was present in HPLC fractions 18 and 19 which had trail following activity but was absent in the neighbouring inactive fractions 17 and 20.

**
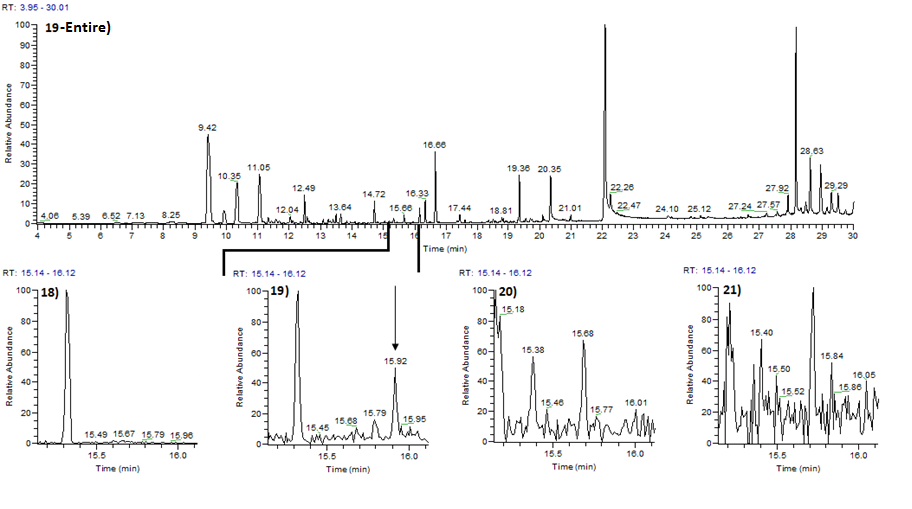
**

**Figure S3**. **GC-MS analyses of HPLC fractions from extracts of headless ants.**

Extracted ion chromatogram for m/z 178 in HPLC fractions numbered 18-21. GC-MS analyses revealed a compound of interest in the most active fraction 19 with a retention time of 15.92 mins and which is marked by the arrow. This compound was absent in HPLC fractions 18 and 21 with weaker activity.
